# Attitude Towards Suicide and Help-Seeking Behavior Among Medical Undergraduates in a Malaysian University

**DOI:** 10.1007/s40596-021-01513-z

**Published:** 2021-08-03

**Authors:** Suzaily Wahab, Nicholas Elam Shah, Sarmeswaran Sivachandran, Izzati Shahruddin, Nik Nor Shaida Ismail, Loushinnah Devi Mohan, Mohammad Rahim Kamaluddin, Azmawati Mohammed Nawi

**Affiliations:** 1 Universiti Kebangsaan Malaysia Medical Centre, Cheras, Kuala Lumpur, Malaysia; 2 Universiti Kebangsaan Malaysia, Bangi, Selangor Malaysia

**Keywords:** Suicide, Attitudes, Help-seeking behavior

## Abstract

**Objective:**

The attitude of medical personnel towards suicide may influence the outcome of suicidal-patients management. This study aimed to determine the attitudes of medical undergraduates towards suicide and its association with their help-seeking behavior.

**Methods:**

A cross-sectional study involving 290 medical undergraduates was conducted in a Malaysian university. The questionnaires on the attitude towards suicide and general help-seeking behavior were used as research instruments.

**Results:**

The mean age of the participants was 22.4 years. Participants who did psychiatry posting indicated a greater tendency to agree on suicide as a way of communication (p = 0.008) than those who did not. Participants previously diagnosed with a psychiatric illness indicated a greater ability to understand and accept suicide (p < 0.001) as well as a greater tendency to agree on the normality of suicide (p = 0.019) than those without a previous diagnosis. Those who attended a suicide prevention program also indicated a greater tendency to agree that loneliness and avoidance could be triggers to suicide (p = 0.037) than those who did not. No correlation was found between the “attitude towards suicide” and “general help-seeking behavior” variable.

**Conclusion:**

Education programs in suicide prevention and management need to be incorporated early into the undergraduate medical curriculum to cultivate a more positive attitude towards suicide and help-seeking behavior.

Suicide has become the second leading cause of death among 15–29-year-olds worldwide [1]. The number of deaths by suicides was close to 800,000 in 2016, with one death every 40 s [2]. In Malaysia, a 2018 estimate showed the annual suicide rate as 5.0–9.9% per 100,000 people [3]. The Malaysian National Health Morbidity Survey in 2015 reported that 29.2% of adults and 12.1% of children suffered from mental illness, with the prevalence of suicide attempts apparently increasing [4]. A previous study in 2009 also reported that the suicide rate among youths (15–24-year-olds) in Malaysia was 1.03 per 100,000 people [5]. Suicide is seen as a great stigma on the family and has legal implications. The most recent available evidence in this respect was from the local suicide registry that was published in 2008. The suicide rate was 1.18 per 100,000 people [6]. However, the reported rate could be underestimated as it only considered medically certified deaths and not the actual number of suicides.

Suicide has become one of the major occupation hazards among physicians and medical students. According to the American Foundation for Suicide Prevention, there is an average of one physician suicide in the USA every day, with the prevalence suicide rate being more than twice that of the general population [7]. Based on a recent meta-analysis study, there were significant numbers of suicide attempts among medical students compared to the general student population. The lifetime and 12-month prevalence of suicide attempts among medical students were 2.19% and 1.64%, respectively [8]. Medical students tend to experience stress caused by many contributing factors, including daily workload, less leisure time, high expectations, and frequent tests that they must pass to sit the final examination. Other than that, owing to the high burdens and demands that medical students face physically and emotionally, burnout is common. Burnout is one of the important factors from which suicidal ideation among medical students could be predicted. A study done in the USA among medical students, residents, and early career physicians found that more than half of them experienced burnout [9]. One local study also showed a high number of medical students experiencing burnout [10]. Apart from work environment that causes burnout, students’ personalities also play a part in contributing to suicidal ideation among them. One study about the association of the personalities of medical students and suicide risk showed that medical students who scored higher in the “stability” personality dimension, which consisted of composure, self-confidence, and tolerance to frustration, were less likely to have suicidal ideation compared to those who scored higher in the “dominance” personality dimension, which consisted of potential, independence, and readiness for conflict [11]. A few studies also stated that the ongoing training process or curriculum could affect the mental health of medical students unintentionally and negatively, with a high frequency of depression, anxiety, and stress [12]. One former study concluded that academic pressure is one of the significant suicide risk factors among Chinese medical students [13]. One study that examined suicidal and general help-seeking behaviors in medical students revealed that they were less likely to seek help or intervention for suicide but prefer to seek informal help because of stigmatizing attitudes [14].

It is well understood that those express about suicide are possibly seeking for help or support [15]. For some people experiencing anxiety, depression, and hopelessness, they may feel that suicide is the only option. Considering the presence of more challenging and more risky environments in the lives of medical students, it is even more crucial to understand how such students will seek help when facing stress or feeling suicidal. This study aimed to investigate the attitudes of medical students towards suicide, and their help-seeking behavior, and factors affecting the two components.

## Methods

A cross-sectional study involving medical undergraduates from year 1 to year 5 was conducted in a public university in Malaysia to determine their attitude towards suicide, and help-seeking behavior, as well as the relation between the two. Stratified random sampling was used to obtain a total of 290 participants. The sample size was calculated using the Power and Sample Size software version 3.1.2 [16]. The female-to-male ratio was taken as 1:1. We selected odd-number names from the name list from which we acquired 58 participants according to the gender ratio from each academic year of study. A participant information sheet was given to each participant who was also briefed on the purpose of the study. The participants’ anonymity was assured to guarantee confidentiality and that no identifiable information was collected. Consent forms were duly filled out by each participant prior to the study. The study was approved by the institution’s Research Ethics Committee. Attitude towards suicide and general help-seeking behavior were assessed by using two questionnaires: the Attitude Towards Suicide (ATTS) questionnaire [17], and the General Help-Seeking Behaviour Questionnaire (GHSQ) [18, 19], respectively.

The ATTS questionnaire was developed to allow a large-scale measurement of attitudes towards suicide [17]. It is scored using a 5-point Likert scale ranging from 1 (strongly disagree) to 5 (strongly agree). For this study, the validated Malay version of the ATTS questionnaire was used [20]. The overall Cronbach’s α score of the adapted Malay version was 0.72 and was proven to demonstrate adequate psychometric properties for use among healthcare personnel in Malaysia. An exploratory factor analysis discovered 11 interpretable factors as “ability to understand and accept suicide” (D1); “suicide among youth and tabooing” (D2); “believability of suicidal threats” (D3); “loneliness and avoidance as suicide triggers” (D4); “judgment and ability to help” (D5); “nature of suicidal ideation” (D6); “acceptability of assisted suicide” (D7); “suicide as communication” (D8); “incomprehensibility” (D9); “normality of suicide” (D10); and “duty to prevent and mental illness” (D11) [20].

The GHSQ was used in this study to examine the psychological help-seeking intentions of the study participants. It used a matrix format with different help-seeking targets in response to two hypothetical scenarios in which a personal-emotional problem and suicidal thoughts were experienced. The authors identified two oblique subscales representing formal and informal help-seeking sources instead of interpreting the questionnaire item by item [21]. Items were measured on a 7-point scale ranging from 1 (extremely unlikely) to 7 (extremely likely). The GHSQ has a Cronbach’s α score of 0.85. Back-to-back translations were performed to obtain the appropriate Malay version for the purpose of this study.

Data collected were analyzed by using Statistical Package for the Social Sciences (SPSS) version 22. Data analysis was performed by using descriptive and inferential statistics to obtain the mean and standard deviation for each of the eight factors measuring the medical undergraduates’ attitudes towards suicide. Statistically significant differences between the independent variables in relation to the dependent variable according to the domains in the ATTS were identified by using analysis of variance (ANOVA) and independent *t* tests. In addition, the Pearson correlation coefficient was used to measure the statistical relation, or association, between the respondents’ attitudes towards suicide and their help-seeking behavior.

## Results

Two hundred and ninety (*N* = 290) medical undergraduates enrolled in this study. The distribution of their enrolment was even (*n* = 58) from year 1 to year 5. The response rate was 100%. The respondents’ mean age was 22.4 years (SD = 1.756) ranging from 19 to 27 years. The participants consisted of 50.3% males (*n* = 146) and 49.7% females (*n* = 144), with only 1% of them as married (*n* = 3) while all the others were single (*n* = 287). Of the participants, 6.2% (*n* = 18) had a psychiatric history, and 7.9% (*n* = 23) had a family history of psychiatric illness. Only 15.2% (*n* = 44) of the participants attended previous formal training/teaching about suicide other than psychiatry posting, and 40% (*n* = 116) of the participants have undergone psychiatry posting.

Table 1 shows the means and standard deviation according to the domains on the ATTS. The participants indicated the greatest tendency to agree on the domain “duty to prevent and mental illness” (D11), with the mean score of 3.84 (SD = 0.785), and “judgment and ability to help” (D5), with the mean score of 3.84 (SD = 0.75). However, the students indicated the weakest tendency to agree on “ability to understand and accept suicide” (D1), with a mean score of 1.850 (SD = 0.72).

**Table 1:** Mean score according to domains of attitude towards suicide

Domain (*N*=290)	Mean (SD)
1) Ability to understand and accept suicide	1.850 (±0.72)
2) Suicide among the young and tabooing	2.176 (±0.88)
3) Believability of suicidal threats	2.595 (±0.81)
4) Loneliness and avoidance	3.401 (±0.81)
5) Judgment and ability to help	3.840 (±0.75)
6) Nature of suicidal ideation	3.489 (±0.68)
7) Acceptability of assisted suicide	2.212 (±1.04)
8) Suicide as communication	2.898 (±0.69)
9) Incomprehensibility	3.172 (±0.79)
10) Normality of suicide	3.195 (±0.99)
11) Duty to prevent and mental illness	3.840 (±0.79)

The students reported a higher help-seeking behavior when they were having suicidal ideation compared to when they were having personal and emotional problems; their mean scores were 3.91 (SD = 1.37) and 3.77 (SD = 1.17), respectively. Students were more likely to seek “informal help” for both personal/emotional problems and suicidal ideation; their mean scores were 4.388 (SD = 1.62) compared to those for “formal help,” which were 3.05 (SD = 1.19) and 3.33 (SD = 1.31) for personal/emotional problems and suicidal ideation, respectively. For both personal and emotional problems, and suicidal ideation, the students were significantly more likely to seek informal help rather than formal help (*p*<0.001).

Among the participants, 42.8% (*n* = 124) preferred to seek informal help from an intimate partner when they had personal and emotional problems while 23.1% (*n* = 67) tended to seek help formally from mental health professionals (Table 2). When experiencing suicidal ideation, 42.4% (*n* = 123) of the students opted to seek informal help from an intimate partner while a smaller percentage (34.5%) (*n* = 100) would seek formal help from mental health professionals (Table 2).

**Table 2:** Percentage of likeliness for medical undergraduates to seek help from different help modalities

*N*=290	GHSQ Personal and emotional problem *N* (%)	GHSQ Suicidal ideation *N* (%)
Informal		
Intimate partner	124 (42.8)	123 (42.4)
Friend	67 (23.1)	86 (29.7)
Parent	134 (46.2)	128 (44.1)
Other relative/family member	67 (23.1)	71 (24.5)
Formal		
Mental health professional	67 (23.1)	100 (34.5)
Phone helpline	23 (7.9)	36 (12.4)
Doctor/GP	53 (18.3)	88 (30.3)
Minister or religious leader	12 (4.1)	22 (7.6)
Nobody	36 (12.4)	30 (10.3)
Someone else	65 (22.4)	71 (24.5)

Among medical undergraduates, the correlation between general help-seeking behavior and attitude towards suicide (Table 3) is weak but positive, that is, between the domains “judgment and ability to help” (D5) (*p* = 0.026, *r* = 0.131) and “incomprehensibility” (D9) (*p* = 0.030, *r* = 0.127) with both informal help-seeking behavior, which means that those who did not find suicide justifiable and did not understand why others chose to end their lives were more likely to seek help through informal means. There is also a weak negative correlation between the domains “ability to understand and accept” (D1) (*p* = 0, *r* = −0.222) and “normality of suicide” (D10) (*p* = 0.041, *r* = −0.120) with both informal help-seeking behavior, which means that those who had a higher understanding of suicide and found it common would seek other means of help rather than informal help. There is a weak positive correlation between the domain “acceptability of assisted suicide” (D7) with formal help-seeking behavior in personal and emotional problems (*p* = 0.005, *r* = 0.163). This means that the more receptive the students were to assisted suicide, the more likely they were to seek help through formal means for personal and emotional problems. There is also a weak negative correlation between “nature of suicidal ideation” (D6) with formal help-seeking behavior in suicidal problems (*p* = 0.004, *r* = −0.167), which means that those who indicated agreement that people who thought about and contemplated suicide constantly tended to ignore any intentions of seeking formal help.

**Table 3:** Means and *p* values of sociodemographic factors and attitude towards suicide for domains 1 to 6

Variables	Ability to understand and accept suicide (D1)		Suicide among young and tabooing (D2)		Believability of suicidal threats (D3)		Loneliness and avoidance as suicide triggers (D4)		Judgment and ability to help (D5)		Nature of suicidal ideation (D6)	
	Mean (SD)	*p* value^b^	Mean (SD)	*p* value^b^	Mean (SD)	*p* value^b^	Mean (SD)	*p* value^b^	Mean (SD)	*p* value^b^	Mean (SD)	*p* value^b^
Age	0.134	**0.023** ^**a**^	−0.06	0.309^a^	−0.028	0.0636^a^	0.109	0.64 ^a^	−0.074	0.208 ^a^	−0.099	0.093 ^a^
Gender												
Male	1.964 (0.747)	**0.007**	2.260 (0.901)	0.101	2.685 (0.85)	0.058	3.397 (0.835)	0.935	3.849 (0.793)	0.837	3.434 (0.683)	0.167
Female	1.735 (0.677)		2.090 (0.856)		2.504 (0.761)		3.405 (0.794)		3.831 (0.714)		3.544 (0.671)	
Year of study												
Pre-psychiatry posting	1.804 (0.862)	0.18	2.204 (0.874)	0.506	2.649 (0.781)	0.162	3.358 (0.834)	0.272	3.885 (0.730)	0.215	3.542 (0.669)	0.099
Post-psychiatry posting	1.920 (0.774)		2.134 (0.895)		2.513 (0.858)		3.466 (0.783)		3.773 (0.786)		3.408 (0.687)	
Family income												
RM 1000–3999	1.831 (0.701)	0.068	2.307 (0.895)	0.149	2.555 (0.839)	0.244	3.373 (0.903)	0.566	3.916 (0.772)	0.080	3.457 (0.685)	0.351
RM 4000–7999	2.011 (0.797)		2.109 (0.863)		2.75 (0.724)		3.432 (0.701)		3.774 (0.712)		3.457 (0.660)	
RM 8000–11,999	1.805 (0.671)		2.012 (0.833)		2.476 (0.806)		3.537 (0.809)		3.61 (0.734)		3.463 (0.703)	
≥ RM 12,000	1.663 (0.645)		2.068 (0.893)		2.545 (0.888)		3.303 (0.736)		3.955 (0.756)		3.659 (0.665)	
Family history of psychiatric												
Yes	2.029 (1.096)	0.411	1.826 (1.124)	**0.047**	2.435 (0.957)	0.327	3.594 (0.791)	0.236	3.58 (0.747)	0.084	3.507 (0.618)	0.89
No	1.835 (0.860)		2.206 (0.853)		2.609 (0.801)		3.385 (0.815)		3.863 (0.751)		3.487 (0.684)	
Diagnosis of psychiatric illness												
Yes	2.574 (1.005)	**0.005**	1.972 (1.156)	0.444	2.889 (1.023)	0.218	3.537 (0.751)	0.465	3.352 (0.881)	**0.004**	3.5 (0.586)	0.941
No	1.802 (0.674)		2.189 (0.861)		2.575 (0.797)		3.392 (0.818)		3.873 (0.735)		3.488 (0.685)	
Previous formal training/teaching of suicide												
Yes	1.727 (0.589)	0.221	1.943 (0.794)	0.057	2.421 (0.889)	0.123	3.636 (0.948)	**0.037**	3.796 (0.698)	0.669	3.401 (0.645)	0.356
No	1.872 (0.741)		2.218 (0.891)		2.626 (0.798)		3.359 (0.782)		3.848 (0.764)		3.504 (0.684)	

Bold indicates significance

^a^Pearson correlation

^b^Student’s *t* test

Analyses for the association between the sociodemographic factors and the attitude towards suicide are shown in Tables 3 and 4. The result shows a weak positive correlation between the students’ age and attitude towards suicide in the domain of “ability to understand and accept suicide” (D1) (*r* = 0.134; *p* = 0.023), “suicide as communication” (D8) (*r* = 0.237; *p* < 0.001), and “normality of suicide” (D10) (*r* = 0.253; *p* < 0.001). A Student’s *t* test between gender and attitude towards suicide shows that males indicated a greater tendency to agree on “ability to understand and accept suicide” (D1) than did females (males = 1.964, females = 1.735; *p* = 0.007). Males were also found to have a greater tendency to be accepting of assisted suicide (D7) than did females (males = 2.39, females = 2.04; *p* = 0.004). There was a statistically significant difference between the participants who underwent psychiatry posting compared to those who did not. Those who completed psychiatric posting indicated a greater tendency to agree on “suicide as a way of communication” (D8) (*p* = 0.008) and “normality of suicide” (D10) (*p* = 0.003) than those who did not. Those without a family history of psychiatric illness indicated a greater tendency to agree on the statement “suicide should not be committed, and it is a taboo” (D2) (*p* = 0.047) than those with such a family history. Participants with a psychiatric history expressed a greater tendency to agree on “ability to understand and accept suicide” (D1) (*p* = 0.005), “normality of suicide” (D10) (*p* = 0.019), and “acceptability of assisted suicide” (D7) (*p* = 0.042) than did those without a psychiatric history. Those without any psychiatric history expressed a greater tendency to agree on suicide not being justifiable and people who are suicidal can be helped: “judgment and ability to help” (D5) (*p* = 0.004) than did those with a psychiatric history. Finally, the medical undergraduates with previous formal training and/or teaching of suicide apart from psychiatry posting expressed a greater tendency to agree on “loneliness and avoidance being triggers to suicide” (D4) (*p*=0.037) than did those who had no such previous formal training.

**Table 4:** Means and *p* values of sociodemographic factors and attitude towards suicide for Domain 7 to 11

Variables	Acceptability of assisted suicide (D7)		Suicide as communication (D8)		Incomprehensibility (D9)		Normality of suicide (D10)		Duty to prevent and mental illness (D11)	
	Mean (SD)	*p* value^b^	Mean (SD)	*p* value^b^	Mean (SD)	*p* value^b^	Mean (SD)	*p* value^b^	Mean (SD)	*p* value^b^
Age	0.093	0.114 ^a^	0.237	**0** ^**a**^	-0.044	0.451 ^a^	0.253	**0** ^**a**^	0.01	0.869 ^a^
Gender										
Male	2.387 (0.986)	**0.004**	2.893 (0.672)	0.903	3.212 (0.828)	0.39	3.230 (0.995)	0.55	3.784 (0.841)	0.227
Female	2.035 (1.065)		2.903 (0.728)		3.132 (0.760)		3.160 (0.989)		3.896 (0.724)	
Year of study										
Pre-psychiatry posting	2.216 (1.042)	0.945	2.812 (0.744)	**0.008**	3.244 (0.841)	0.051	3.055 (1.035)	**0.003**	3.862 (0.748)	0.553
Post-psychiatry posting	2.207 (1.041)		3.026 (0.608)		3.065 (0.709)		3.405 (0.884)		3.806 (0.841)	
Family income										
RM 1000–3999	2.236 (1.039)	0.564	2.866 (0.733)	0.855	3.256 (0.789)	0.292	3.11 (1.010)	0.263	3.823 (0.803)	0.850
RM 4000–7999	2.308 (1.137)		2.94 (0.621)		3.179 (0.721)		3.378 (0.916)		3.853 (0.717)	
RM 8000–11,999	2.049 (0.886)		2.943 (0.730)		3.049 (0.797)		3.098 (1.068)		3.927 (0.703)	
≥ RM 12,000	2.125 (1.001)		2.871 (0.720)		3.034 (0.918)		3.205 (0.978)		3.784 (0.930)	
Family history of psychiatric										
Yes	2.043 (1.296)	0.515	3.000 (0.752)	0.466	2.978 (0.923)	0.222	3.022 (1.112)	0.384	3.848 (0.745)	0.959
No	2.227 (1.016)		2.889 (0.696)		3.189 (0.782)		3.21 (0.980)		3.839 (0.790)	
Diagnosis of psychiatric illness										
Yes	2.694 (1.016)	**0.042**	3.185 (0.607)	0.072	2.972 (0.606)	0.27	3.722 (0.927)	**0.019**	3.778 (0.691)	0.731
No	2.18 (1.035)		2.879 (0.702)		3.186 (0.804)		3.16 (0.987)		3.844 (0.792)	
Previous formal training/teaching of suicide										
Yes	3.341 (1.082)	0.373	2.955 (0.703)	0.559	3.068 (0.752)	0.346	3.227 (1.097)	0.814	3.921 (0.889)	0.46
No	2.189 (1.032)		2.888 (0.700)		3.191 (0.802)		3.189 (0.973)		3.825 (0.767)	

Bold indicates significance

^a^Pearson correlation

^b^Student’s *t* test

## Discussion

Our study reveals that the participants had the greatest propensity to agree on the domain “duty to prevent mental illness” and “judgment and ability to help.” This shows that the participants had a particularly strong belief that suicidal persons can be helped, that suicide can never be justified, and that everyone is responsible for preventing suicide. The students also strongly agreed that suicide is associated with mental illness. In contrast, they indicated the greatest tendency to disagree on the domain “ability to understand and accept suicide,” which reflects their disagreement with respect to the idea of a person having the right to take one’s own life even as a way to escape from problems. This can be understood in the context of Malaysia being a country with a Muslim-majority population that does not conform to suicidal behavior, and that sees it as a major sin. One systematic review study has shown that religion is a protective factor for suicidal behavior. A religious country has suicidal rates that are lower than those of a secular country, and individuals who are committed to their religion and often engaged with their religious community have the lowest tendency to end their lives [22]. While all major religions discourage suicide, one study showed that Muslims have a suicide rate that is lower than that of other religions [23]. Besides religiosity, cultural factors play an important role in the display of suicidal behavior in society. Human behavior can be influenced by cultural factors and the effect can be either protective or destructive tendencies with regard to suicide [24]. While variations of suicidal behavior can be seen across regions and countries, such as those of eastern communities (Southeast Asia), that consider suicide a taboo [25], the difference in ethnicities in a country also shows a variation in suicidal behavior. In Malaysia, Indians and Chinese reported high percentages of suicidal cases compared to Malay, which is the major ethnic group [24], in this country where all three ethnic groups mentioned practice different cultures.

The findings of the study showed that medical undergraduates’ attitudes towards suicide were generally affected by age, gender, family history of psychiatric illness, previous diagnosis of psychiatric illness, previous formal training or teaching on suicide, and whether they received psychiatry teaching. On the contrary, marital status and family income were not found to influence the attitudes of the participants.

More senior students were more likely to accept suicide, which means that they had higher tendency to agree about the right for suicide and could understand the need to take one’s own life. Other than that, they also regard suicide as a cry for help, and they were more likely to agree that suicide is normal. Such attitudes of understanding suicide can be understood possibly because of increased knowledge and exposure in handling suicidal patients that were obtained by senior students during their clinical posting. Comparison among gender revealed that attitudes towards suicide among male and female medical undergraduates were almost similar except for the “ability to understand and accept suicide” domain, which was more predominantly seen in males. This contrasts with a finding from a previous study that female health personnel were more understanding to those who were suicidal [26].

The findings of the present study also show that female medical undergraduates indicated a greater tendency to agree on the domain “acceptability of assisted suicide” than did males. This differs from the finding of a study conducted with respect to Poland in which male undergraduates were found to be more willing to make the decision to perform assisted suicide [27]. A study in Malaysia that explored medical undergraduates’ perceptions on assisted suicide revealed that 45.5% of the participants were against it, and 16.5% of them indicated agreement that everyone has the right to choose [28]. However, regarding the legality of assisted suicide, it is crucial to note that, with certain exceptions, it is prohibited by law in Malaysia [29].

Individuals with a family history of psychiatric illness reported that they were more likely to view suicide or suicidal persons differently than were those with no family history of psychiatric illness. This study reveals that participants with a family history of psychiatric illness indicated a weaker tendency than that of those without such history to agree that “young people should not commit suicide as they have everything they need to live” and “suicide is a taboo in the society” as assessed in D2 (suicide among the young and tabooing). However, the findings of the research revealed that students having a past psychiatric diagnosis indicated a greater tendency to agree on suicidal behavior, and believed that anyone can end their own life and that suicide can be assisted. In addition, these students had a weaker tendency to agree that suicide can be helped, that suicide cannot be justified, and that suicide is the worst thing someone can do to their family. This pattern of thinking could be influenced by the state of their mental health at the time of the study as there had been no assessment of their state of psychiatric illness before they participated in the study.

Medical undergraduate students will naturally gain knowledge that influences their attitudes during their studies. Those who had previous formal teaching about suicide expressed a greater tendency to agree that loneliness and avoidance can be the causes of suicide than did those without such teaching. A different study has shown that in various populations in various countries, loneliness has been identified as a factor closely related to thoughts of ending and attempting to end their lives [30]. A similar result has been described in a large-scale community-based study of 5,423 middle school adolescents in Houston, USA. The study showed that feelings of loneliness and isolation could be used to predict suicidal thoughts [31].

Under the present study, the medical faculty incorporated an 8-week psychiatry posting into the curriculum during the fourth year of medical school. This includes a discussion of a suicide module that emphasizes the identification of suicidal symptoms, and suicide risk assessment as well as suicide prevention and management. The teaching takes place in the form of lectures, small group discussions, and tutorials, as well as clinical exposure with real patients in the ward and clinics. The findings of this study show that students who obtained higher scores in two domains, i.e. “normality of suicide” and “suicide as a way of communication,” also have experience in psychiatry posting that influenced their attitudes towards suicidality. This means, those students agreed suicidal act is something normal which can be understood, and people who had the intention to take their own lives are trying to seek for help. This attitude towards suicide could be influenced by their exposure and experiences with suicidal patients during psychiatry posting. In view of this, psychiatry posting provides an important platform to equip these future doctors with education and skills on suicide prevention and management. Researchers of a previous study on attitudes towards suicide in Hong Kong found that the incorporation of a suicide-prevention training module in the medical curriculum had a positive influence on understanding suicide in terms of reducing the score for negative appraisal, stigmatization, and fatalism with respect to suicide [32]. In the same context, the suicide-prevention programs among university students helped to improve suicide knowledge, prevention, and the likelihood of one person asking another person about suicide [33]. This positive outcome is not limited to medical undergraduates as it also applies to nurses who received training in suicide management. Trained nurses were reported to have a strong capability to prevent potential suicide [34] and have improved perceived knowledge, self-efficacy, and willingness to assist suicidal patients [35].

Aligned with findings from another study [36], our findings show that medical undergraduates preferred to seek informal help (from a spouse, friend, or parents) rather than formal help (from doctors, helpline, religious personnel, or other professionals) when dealing with personal and emotional problems as well as suicidal ideation. One recent cross-sectional study found that support from family and friends can protect an adolescent from having suicidal ideation [37]. The preference for informal help-seeking could be due to the strong stigma and fear of being criticized by peers or supervisors, which served as a barrier to treatment for mental health issues [38]. In addition, there may also be the fear that their future medical career might be jeopardized once they are diagnosed with a psychiatric illness [39]. It is worth noting that personal stigma was significantly associated with a lower likelihood of a perceived need for help, use of psychotropic medication, and use of therapy or counseling [40].

Our study is not without limitations. The fact that it was conducted in a single medical school and used the cross-sectional method limits its generalizability. In addition, this study was performed in a country that has a high proportion of Muslims where suicide is seen as a huge taboo. Therefore, the results may not reflect the actual findings as regards the general population. Further, the study did not consider religion and race information although these two variables can significantly affect the suicidal behavior among the participants. Finally, details about the students’ emotional state and their mental health status at the time of the study were not obtained. It is believed that these details could also influence their perspective on suicide and their general help-seeking behavior.

In conclusion, the study was able to provide some insight into understanding the future medical practitioners’ general attitude towards suicide. It also outlines their help-seeking behavior, which is important in planning suitable interventions to avoid negative mental health outcomes. The study also highlights the more positive attitudes of the medical students towards suicide as they received earlier training in, and exposure to, suicide prevention and management during their fourth year psychiatric posting. As for the younger medical undergraduates particularly year 1 to year 3, it is imperative to expose them to information regarding suicide and suicide prevention by hosting events such as Suicide Awareness Month or conducting seminars on this topic. Focus on mental health literacy by introducing short lessons on recognizing mental health issues among younger medical undergraduates is another possibility to overcome the gap of information between them and their older counterparts. Emphasis should be placed on stress management and on mitigating awareness regarding mood symptoms and depression, which are very closely linked to suicidal behavior. Mental health literacy should also cover the skill of dealing with transference and countertransference when attending to suicidal patients, and the awareness of the importance of being non-judgmental towards patients harboring suicidal thoughts or who may have attempted suicide. Moreover, resources to support mental well-being of students should also be provided via counseling services in the faculty and practiced in a non-stigmatizing manner. The implementation of all these measures may hopefully improve mental health outcomes and suicidality in medical students.
